# Topping-off surgery vs posterior lumbar interbody fusion for degenerative lumbar disease: a comparative study of clinical efficacy and adjacent segment degeneration

**DOI:** 10.1186/s13018-019-1245-3

**Published:** 2019-06-28

**Authors:** Dongyue Li, Yong Hai, Xianglong Meng, Jincai Yang, Peng Yin

**Affiliations:** Orthopaedic Department, Beijing Chaoyang Hospital Affiliated to Capital Medical University, Beijing, 100020 China

**Keywords:** Topping-off, Coflex, Posterior lumbar interbody fusion (PLIF), Degenerative lumbar disease (DLD), Clinical efficacy, Adjacent segment degeneration (ASD)

## Abstract

**Background:**

Studies have shown that adjacent segment degeneration (ASD) is a common complication after posterior lumbar interbody fusion (PLIF), even a second surgery is required for some patients. It remains unclear whether the non-fusion surgery can relieve ASD. Therefore, this study aims to investigate the clinical outcomes of Topping-off surgery (fusion combined with Coflex) and PLIF for degenerative lumbar disease (DLD) and the efficacy on preventing ASD.

**Method:**

A retrospective analysis was performed on the clinical data of 99 patients with DLD from January 2011 to December 2014, who were performed by Topping-off surgery (L4–5 PLIF + L3–4 Coflex, *n* = 45) or PLIF (L3–5 PLIF, *n* = 54). All patients included in the analysis had a minimum of 3 years of follow-up. Clinical data were used to assess the clinical efficacy, and radiographic parameters were measured for evaluation of the incidence of ASD.

**Results:**

The mean ages of Topping-off group and PLIF group were 53.5 and 65.7 years old, respectively (*P* < 0.05). The surgical time, intraoperative blood loss, Oswestry disability index (ODI), and visual analog scale (VAS) were significantly different between the two groups (*P* < 0.05). Intervertebral mobility (L2-L3) of the Topping-off group was not changed significantly at 3 years after surgery than before (*P* > 0.05), while that of PLIF group was increased considerably (*P* < 0.05). As to intergroup comparison, intervertebral mobility (L2-L3) of Topping-off group was superior to those of the PLIF group (*P* < 0.05). Surprisingly, there was no significant difference in the general adjacent segment mobility (GASM) at L2–4 of the Topping-off group and intervertebral mobility (L2–L3) of PLIF group at 3 years after surgery (*P* > 0.05). Lumbar MRI at three post-operative years indicated that the modified Pfirrman grading of disc (L2–L3) in the Topping-off group was much better than that of the PLIF group (*P* < 0.05).

**Conclusion:**

This study showed that Topping-off surgery had the benefits of less invasiveness, less bleeding, and comparable clinical efficacy as PLIF for DLD. The segment with Coflex insertion undertook part of the mobility and stress in the proximal lumbar spine, which is conducive to alleviating ASD.

## Background

Posterior lumbar interbody fusion (PLIF) is the most common surgery for degenerative lumbar disease (DLD), such as lumbar disc herniation and lumbar spinal stenosis. However, PLIF achieves the clinical efficacy at the expense of mobility of the affected lumbar spine segments, increasing the load to adjacent segments and accelerating degenerative disc disease and facet joint degeneration, which further increases the risk of adjacent segment degeneration (ASD) [1–4]. The incidence of ASD, following open or MIS lumbar instrumented fusions, ranged up to 30% [5]. Some scholars [1–3, 6–9] have found that the risk of ASD is related to various factors, including advanced age, increased body mass index (BMI), cross-sectional area of paraspinal muscles, pre-existing degeneration of adjacent discs, facet injury or tropism, the length of lumbar fixation, lower sacral slope, and post-operative sagittal alignment.

A non-fusion interspinous device, such as Coflex, is a known alternative technique to PLIF [10]. It preserves a certain degree of vertebral activity, which reduces the incidence of ASD [4, 10, 11]. Previously biomechanical and clinical literatures show that Coflex is a safe and effective treatment for DLD [11, 12]. Topping-off surgery (PLIF combined with dynamic stabilization of interspinous space of Coflex in the superior segment) is designed to prevent ASD [13, 14]. The protective effect of this approach was provided by Coflex insertion [4, 11], and few studies are concerned with the clinical mechanism of the protective effect on the superior adjacent segment. Thus, the purpose of this retrospective study was to compare the clinical outcomes and radiographic parameters between Topping-off surgery and PLIF for DLD. In addition, we should investigate the working mechanism about the degeneration of superior adjacent segment from the radiographs.

## Methods

### Clinical data

From January 2011 to December 2014, 99 patients with DLD at L3–L5 were recruited. All patients had lower back pain before surgery, which was combined with intermittent claudication or nerve root compression and unresponsiveness to conservative treatment for at least 6 months. Lumbar spine MRI, CT scan, and X-ray in standing anteroposterior/lateral and flexion/extension views were taken before surgery. Combining with the clinical symptoms and physical examination, the responsible segments were identified as L3–L5. When the possibility of L3 nerve root damage could not be excluded, we will choose L3 nerve root block before surgery. After determining L2–3 as a non-responsible segment, these cases would be included. The following conditions were excluded: degenerative lumbar scoliosis or kyphosis, lumbar spine fracture, spondylolisthesis at L3–L4 of grade II and above, severe osteoporosis, and history of lumbar spine surgery. Those without spondylolisthesis or isthmus fracture at L2–L3 were selected.

### Surgical procedures

#### PLIF

The patients took a prone position after general anesthesia. Disinfection and draping were performed conventionally. The posterior middle approach was adopted, and subperiosteal stripping was performed until reaching bilateral facet joints. Attention was given to protect the facet joint capsule. Pedicle screws were inserted bilaterally at L3–L5 for internal fixation. Interlaminar fenestration or total laminectomy was performed bilaterally at L4–L5 for decompression. Interlaminar fenestration was performed bilaterally for decompression at L3–L4, with preservation of the lateral 1/2 of the facet joint. Similar scope of bone resection was ensured for the segments with Coflex resection as in the Topping-off surgery. The thick hypertrophic ligamentum flava were resected, and the nerve root was completely loosened until the outlet. The intervertebral disc was resected, and cartilaginous end-plate scrapped. After the loosening of the nerve root was inspected, intervertebral bone grafting was performed and cage of the appropriate size was inserted.

#### Topping-off surgery

The same procedures were performed to expose the target area and to manipulate the segments at L4–L5 as PLIF. Supraspinous ligament of sufficient width was preserved, and the integrity of vertebral spinous process at L4 was protected. Coflex was inserted to L3–L4 with interlaminar fenestration for decompression. The medial superior and inferior articular processes were partially resected, while the lateral aspects were preserved. At least 50% of the facet joint was preserved. Part of the ligamentum flava was resected. The nerve canal root was enlarged, and the protruding nucleus pulposus was removed. After nerve root loosening was inspected, the supraspinous ligament was isolated between the superior and inferior spinous processes and tracted away. The superior interspinal ligament and ligamentum flavum were resected to expose the posterior dural sac. The upper and lower edges of the spinous process were trimmed, and the test mode was implanted. After adjusting to appropriate tightness, Coflex of the corresponding size was inserted between the spinous processes, with its top at about 2 mm from the dural sac. Reduction was performed for the interspinal ligament, and a hole was drilled in the spinous process. Suture was performed for in situ immobilization.

The implant position was checked by intraoperative X-ray, and the incision was thoroughly washed. The drainage tube was indwelled, and the suture was performed layer by layer. The patients received conventional postoperative antibiotics for 48 h and could leave the bed and stand after the drainage tube was removed 3 days later. The patients wore lumbar support belt for 2–3 months after discharge.

### Efficacy evaluation and radiographic assessment

**Fig. 1:**
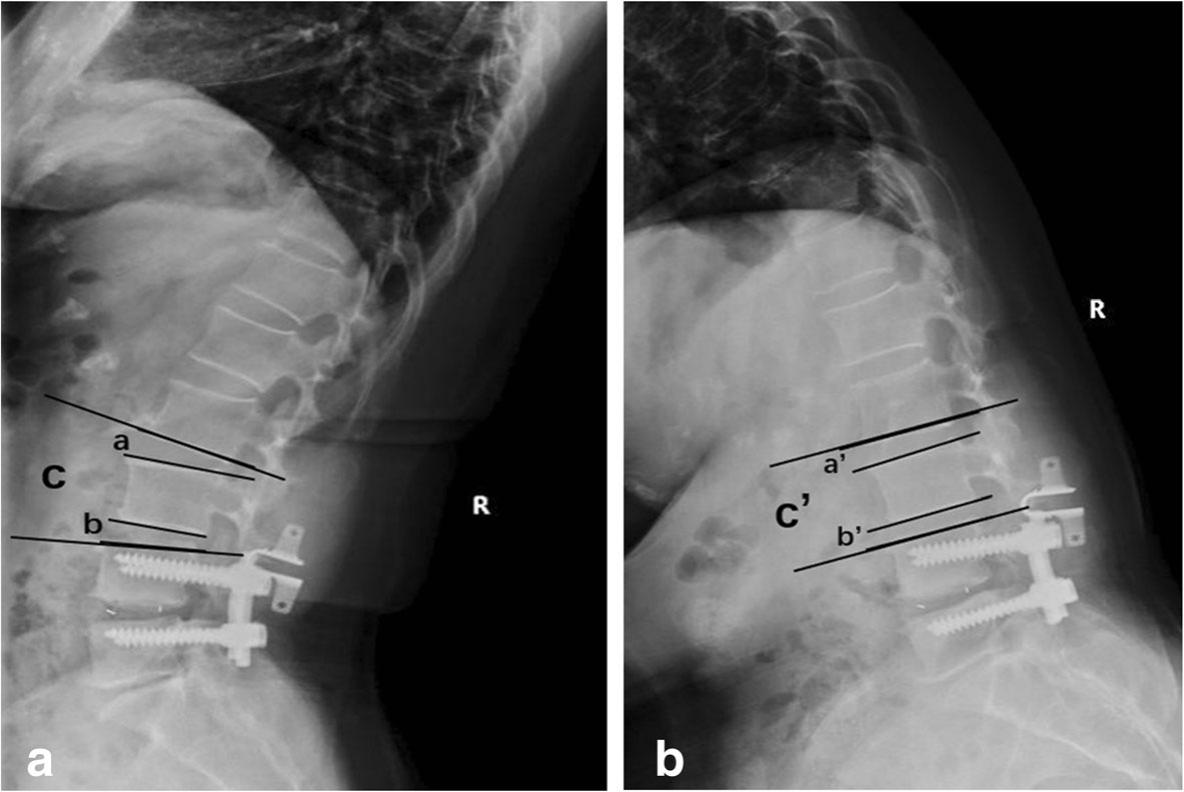
Lateral flexion and extension lumbar X-ray showing radiological indices studied. Adjacent intervertebral mobility (L2–L3) = extension angle (a)–flexion angle (a’). Intervertebral mobility (L3–4) with Coflex insertion = extension angle (b)–flexion angle (b’). General adjacent segment mobility (GASM) at L2–4 = extension angle (c)–flexion angle (c’)

All patients received regular reexaminations at outpatient clinic. VAS and ODI scores for lower back/leg pain were recorded before surgery and at 3 years after surgery. The following indicators were measured: adjacent intervertebral mobility (L2–L3) by X-ray of the lumbar spine in standing flexion and extension views, intervertebral mobility of the segments (L3–4) with Coflex insertion, modified Pfirrmman grading of disc (L2–3), and general adjacent segment mobility (GASM) at L2–4 in the Topping-off group at 3 years after surgery. The new name of GASM means sum of intervertebral mobility at L3–L4 with Coflex insertion and that of the superior L2–L3. (Fig. 1).

Radiographic parameters were measured by using the following method: intervertebral mobility: the angle of inclusion between the extended lines of the superior and inferior end-plates at the corresponding intervertebral space was measured by X-ray of the lumbar spine in standing flexion and extension views, and the difference between the flexion and extension views was the intervertebral mobility; modified Pfirrmann grading of disc [15]: based on sagittal T2-weighted MRI image of the lumbar spine, the modified Pfirrmman grading for lumbar intervertebral disc degeneration was performed at L2–L3 levels before surgery and at 3 years after surgery.

Radiographic criteria for ASD were as follows: (1) intervertebral mobility for adjacent segment > 10° from X-ray taken in flexion and extension views [15]; (2) an increase in the modified Pfirrmann grading of disc [16].

### Statistical analysis

All statistical analyses were performed using software SPSS 13.0. Measurements were reported as ± standard deviation. Paired *t* test was used for comparison of preoperative and postoperative scores and radiographic parameters within the same group. Independent-samples *t* test was used for intergroup comparison. Chi-square test was used to determine if two categorical variables were independent. Variable data on modified Pfirrmann grading for lumbar intervertebral disc degeneration were compared by using the rank-sum test. *P* < 0.05 indicated significant difference.

## Results

### Surgical results

Surgeries were successfully performed for all 99 cases. There were 45 patients receiving Topping-off surgery (PLIF at L4–L5 + Coflex insertion at L3–L4), with 21 males and 24 females (aged 46–59 years; average age, 53.5 years), and 54 patients receiving PLIF at L3–L4, with 25 males and 29 females (aged 60–75 years; average age, 65.7 years). Both two groups were measured for lumbar spine bone mineral density before surgery, and none of them had severe osteoporosis (*T* value > − 2.5).

The surgical time was 120–180 min for the Topping-off group, with an average of 147.3 ± 29.3 min; the intraoperative blood loss was 150–320 ml, with an average of 220.3 ± 57.4 ml. The surgical time was 160–240 min for the PLIF group, with an average of 208.2 ± 37.6 min; the intraoperative blood loss was 210–580 ml, with an average of 377.6 ± 83.8 ml. The surgical time and intraoperative blood loss of the Topping-off group significantly decreased compared with the PLIF group (*t* = 11.75, *t* = 18.57, *P* < 0.05). There were no complications of spinal dural mater rupture and nerve injury during surgery.

### Clinical outcome

The two groups of patients were comparable in terms of age (*P* < 0.05), gender (*P* > 0.05), and bone mineral density (*P* > 0.05). In the Topping-off group, one case had intraspinal hematoma after surgery; in the PLIF group, one case had subcutaneous incision infection and another case intraspinal hematoma. Three cases achieved satisfactory outcomes after symptomatic treatment. All patients were followed up for 47.2 ± 8.8 months (36–72 months) after surgery. During the follow-up, one patient in the PLIF group reported symptoms due to herniated disc at L2–L3 at 48 months after surgery and received a revision surgery. The symptoms were significantly improved after the second surgery. Both two groups had a significant improvement in VAS and ODI scores for lower back/leg pain at 3 years after surgery than before (*P* < 0.05). But there was no significant difference in the pairwise comparison (*P* > 0.05) (Table 1).

**Table 1:** Comparison of VAS and ODI scores (±SD)

Group	VAS for lower back pain		VAS for leg pain		ODI	
	Before surgery	3 years after surgery	Before surgery	3 years after surgery	Before surgery	3 years after surgery
Topping-off	6.32 ± 1.74	1.91 ± 0.73	4.78 ± 1.22	1.50 ± 0.61	48.09 ± 9.61	13.65 ± 4.14
PLIF	6.49 ± 1.90	2.14 ± 0.91	5.11 ± 1.48	1.63 ± 0.63	50.54 ± 11.21	14.22 ± 5.03
*t*	0.385	0.323	0.271	0.308	1.617	0.676
*P*	> 0.05	> 0.05	> 0.05	> 0.05	> 0.05	> 0.05

*P* < 0.01, before surgery and at 3 years after surgery

### Radiographic parameters

#### X-ray

Topping-off group: Adjacent intervertebral mobility (L2–L3) was not significantly changed after surgery (*P* > 0.05), and that at L3–L4 with Coflex insertion was decreased than before (*P* < 0.05). GASM at L2–4 (sum of intervertebral mobility at L3–L4 and L2–L3) was above 10° in 3 cases; however, the intervertebral mobility at either L3–L4 or L2–L3 was below 10°.

PLIF group: Adjacent intervertebral mobility (L2-L3) was significantly higher after surgery than before (*P* < 0.05), and it was above 10° in 2 patients.

Intergroup comparison: The two groups had no significant difference in intervertebral mobility (L2–L3) before surgery (*P* > 0.05). After surgery, it was lower in the Topping-off group than in the PLIF group (*P* < 0.05). Surprisingly, at 3 years after surgery, GASM (L2–4) was not significantly different between the two groups (*P* > 0.05) (Table 2).

**Table 2:** Radiographic parameters (±SD)

Group	Intervertebral mobility at L2–3		Intervertebral mobility at L3–4		General adjacent segment mobility (GASM)
	Before surgery	3 years after surgery	Before surgery	3 years after surgery	3 years after surgery
Topping-off	4.11 ± 1.52^a^	4.30 ± 1.71^a^	4.42 ± 1.91^b^	3.03 ± 1.33^b^	7.19 ± 2.31
PLIF	4.29 ± 1.81^b^	7.03 ± 2.14^b^	–	–	7.03 ± 2.14*
*t*	1.364	31.782	–	–	0.721
*P*	> 0.05	< 0.01	–	–	> 0.05

^a^*P*>0.05, ^b^*P*<0.05, comparison before surgery and at 3 years after surgery

*It is the intervertebral mobility at L2–L3

#### Lumbar MRI

The two groups showed no significant difference in modified Pfirrmann grade of disc at L2–L3 before surgery (*P* > 0.05). At 3 years after surgery, the modified Pfirrmann grade of disc was increased by 1 grade in 2 cases of the Topping-off group (4.44%).

In contrast, disc degeneration was more severe in the PLIF group, with increased Pfirrmann grade in 14 cases (25.93%) (including 2 cases with intervertebral mobility > 10°). Among them, 11 cases had an increase by 1 grade, 2 cases 2 grade, and 1 case 3 grade (this patient received a revision surgery). The difference was of statistical significance between the groups (*P* < 0.05).

**Fig. 2:**
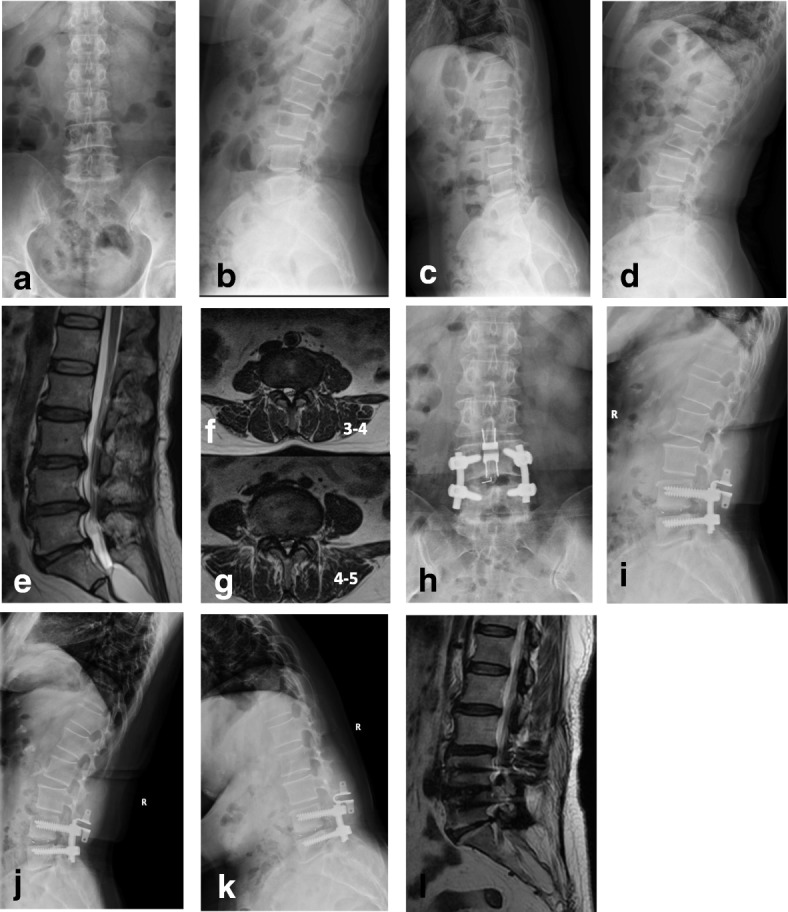
A 44-year-old, female, patient complained of lower back pain and pain in the left lower limb for 8 years, with aggravation for 6 months. This patient had spinal canal stenosis at L3–L5 and received Topping-off surgery. **a–d** X-ray for the lumbar spine in the anteroposterior/lateral and flexion/extension views before surgery; intervertebral mobility 3.9° at L2–L3 and 4.2° at L3–L4. **e–g** Lumbar MRI scan before surgery indicated spinal canal stenosis at L3–L5, with modified Pfirrmann grade of disc 4 at L2–L3. **h–k** X-ray for the lumbar spine in the anteroposterior/lateral and flexion/extension views at 36 months after surgery. There was no significant change in the intervertebral mobility at L2–L3, which was 4.2° after surgery; the intervertebral mobility at L3–L4 was decreased to 2.9° after surgery. **l** MRI scan at 36 months after surgery indicated that the modified Pfirrmann grade of disc was still 4 at L2–L3

**Fig. 3:**
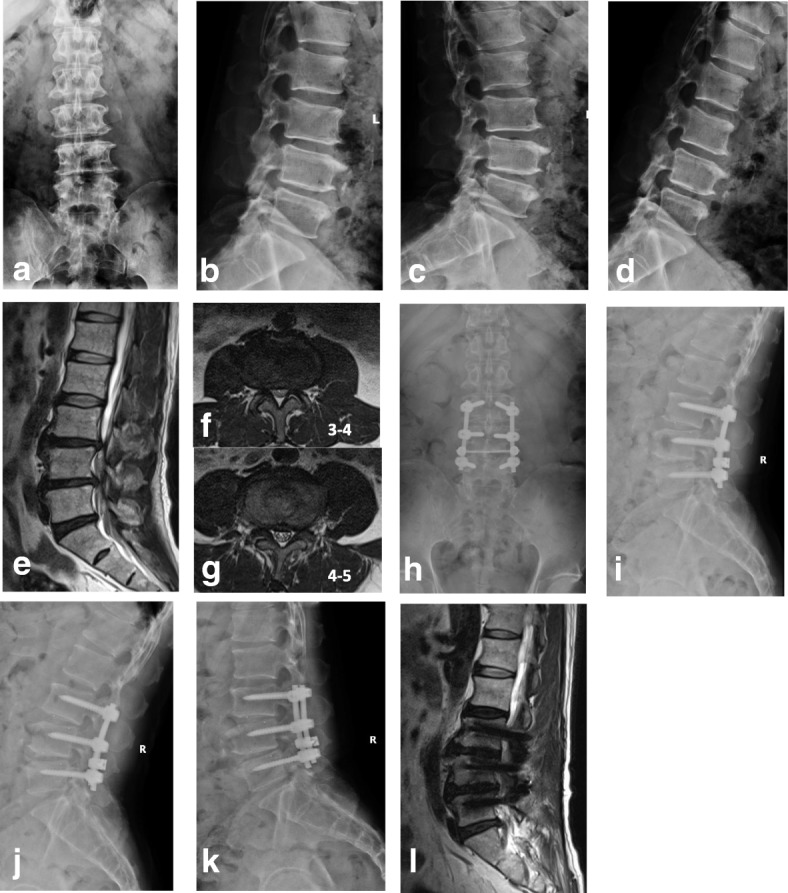
A 66-year-old, male, patient complained of lower back pain and pain in bilateral lower limbs for 2 years and aggravation for 7 months. There was spinal canal stenosis at L3–L5, and the patient received PLIF at L3–L5. **a–d** X-ray for the lumbar spine in the anteroposterior/lateral and flexion/extension views before surgery; intervertebral mobility 4.3° at L2-L3. **e–g** Lumbar MRI scan before surgery indicated spinal canal stenosis at L3–L4 and L4–L5, with modified Pfirrmann grade of disc 3 at L2–L3; **h–k** X-ray for the lumbar spine in the anteroposterior/lateral and flexion/extension views at 36 months after surgery. The intervertebral mobility at L2–L3 was increased significantly to 7.3° after surgery. **l** MRI scan at 36 months after surgery indicated that the modified Pfirrmann grade of disc was increased to grade 4 at L2–L3

For description of representative cases, see Figs. 2 and 3.

## Discussion

PLIF is the major treatment for DLD (e.g., spinal canal stenosis, lumbar disc herniation), though usually the expense of spinal mobility at the operated segment, which causes a compensatory increase in the mobility of adjacent segments and increases the risk of ASD [1–3, 6–9]. Nancy [5] reported that the incidence of ASD after PLIF is even as high as 30%, which is similar to that of ASD after PLIF in our study. The biomechanics study [12, 17] has shown that due to the affected segment fixed, the mobility and stress of the adjacent segment increase as a result, leading to an apparent increase in the slipping and flexion and extension mobility than in the normal conditions. This finally leads to an increased risk of ASD. According to Alentado et al. [3], 9% of the patients develop symptomatic ASD within 2 years after PLIF and require a second surgery. Therefore, preventing ASD is of high clinical significance.

Topping-off surgery, or PLIF combined with Coflex insertion in the superior segment, has been applied to DLD. Some researchers have shown that a similar effect is achieved for the segment with Coflex insertion as with PLIF [4, 11–13, 18–20]. In our study, VAS and ODI scores were improved significantly at 3 years after surgery than before in both groups (*P* < 0.05). That is to say, Topping-off and PLIF can both relieve the symptoms. The former had a significant reduction in surgical time and intraoperative blood loss than the latter (*P* < 0.05). This is probably because there is no need to expose the intact facet joint and transverse process for Coflex insertion, but exposure of medial 1/2 of the facet joint was required. Moreover, no pedicle screw for internal fixation and bone graft in intervertebral space or between the transverse processes is needed, and that explains the reduction in surgical time and intraoperative blood loss. Patients with DLD are usually of an elder age. In the present study, the average age of patients in the Topping-off group was 53.5 years old and that of the PLIF group was 65.3 years old. These patients are more likely to have underlying diseases, such as hypertension and diabetes. Thus, less surgical time and intraoperative blood loss will contribute to safety and postoperative recovery. When Topping-off surgery was performed, severe intervertebral disk degeneration, instability, slippage, or isthmus fracture should be carefully watched for segments with Coflex insertion, so as to prevent failure of the implant.

Compared with fixed lumbar vertebrae after PLIF, the segment with Coflex insertion maintains a certain mobility. People have been working until 60 years old in our country, and it is very important for them to reserve lumbar activity. In this study, Topping-off surgery was chosen before 60 years old in order to remain maximize lumbar motion. In contrast, we chose PLIF when patients were older than 60 years old. Due to partial activity of the segment of Coflex insertion, there was a reduction in spinal stress to the superior adjacent segment, which is conducive to reducing the risk of ASD [4, 11, 18–23]. Biomechanical studies [10, 12, 17] have shown that the Coflex device exhibits excellent compression stiffness and tensile stiffness, thus providing good stability to the lumbar spine. In our study, Topping-off surgery involves partial resection of ligamentum flavum and articular process, enlargement of the nerve root canal, removal of the herniated disk or nucleus pulposus for the decompression of dural mater, and loosening of the nerve root. Special attention should be paid to protecting the lateral half of the articular process in the segment of Coflex insertion. The aim was to preserve the stable structure of the posterior spine as far as possible. With partial preservation of flexion and extension mobility, the spinal stress will not be excessively concentrated in the superior adjacent intervertebral space, which is conducive to protecting adjacent intervertebral space [4, 12, 17].

In healthy subjects, all segments are involved in the overall flexion and extension mobility of the lumbar spine. With any segment immobilized, the other segments will have a compensatory increment of mobility [1, 19]. In our study, we observed the changes in adjacent segment mobility by X-ray in the standing flexion and extension views. For the PLIF group, after complete restriction of mobility at L3–L5, the mobility of L2–L3 at 3 years after surgery was increased significantly than before (*P* < 0.05). For the Topping-off group, with the complete restriction of mobility at L4–L5, Coflex insertion at L3–L4 had a certain reduction in mobility than before (*P* < 0.05), but part of the mobility was still preserved. For the decline of mobility, this is because the inserted Coflex pushes aside the spinous process and stabilizes the posterior column, thus increasing the foraminal height [14] and partially preventing posterior stretch of the spine. So Coflex insertion has a much less impact on spinal flexion [4, 12]. Elastic immobilization ensures that Coflex has a certain mobility [10] after its insertion. Because of the buffer provided by Coflex, the superior L2–L3 preserved similar mobility as before (*P* > 0.05). Our study indicated that the intervertebral mobility (L2–L3) in the Topping-off group was much lower than that with PLIF (*P* < 0.05), which was suggestive of the protective effect from the Coflex to the adjacent segment at L2–L3.

Surprisingly, we observed no significant difference between GASM in the Topping-off group and adjacent intervertebral mobility (L2–L3) in the PLIF group (*P* > 0.05). The reason is probably that the original intervertebral mobility of the adjacent segment at L2–L3 if L3–5 was fusion was shared by both L3–L4 with Coflex insertion and the adjacent segment at L2–L3 if Topping off surgery was performed, thus reducing the risk of ASD at L2–L3. The intervertebral mobility (L2–L3) was above 10° in 2 cases from the PLIF group, which satisfied the criteria for ASD on X-ray. Three cases from the Topping-off group had GASM above 10°, but the mobility was shared by two segments, with each below 10°, which did not meet the criteria for ASD on X-ray. To more clearly detect intervertebral disc degeneration after surgery, lumbar MRI scan was performed for L2–L3 at 3 years after surgery. As expected, the incidence of ASD in the PLIF group, which was similar to the results reported in the literatures [5, 7–9], was much higher than that of the Topping-off group (25.93% vs 4.44%, *P* < 0.05). The reason is probably that the inserted Coflex partially undertook the mobility and stress of the proximal lumbar spine, which reduced the incidence rate of ASD.

## Conclusion

Topping-off surgery, a hybrid surgery of lumbar fusion and dynamic stabilization, achieved similar clinical efficacy for DLD as PLIF. Moreover, the former caused less surgical trauma and bleeding and decreased the risk of ASD [4, 12, 13, 20–23]. We considered that the segment with Coflex insertion retained some of the activity and shared the spinal stress. However, our study had the following limitations: the patients were not randomly selected, the duration of follow-up was short, and the sample size was limited. The clinical efficacy and the incidence of ASD of Topping-off surgery for DLD remain to be verified by trials with a larger sample size and longer follow-up.

## Data Availability

All data used and analyzed during this study are available from the corresponding author upon reasonable request.

## Abbreviations

ASD: Adjacent segment degeneration
DLD: Degenerative lumbar disease
GASM: General adjacent segment mobility
ODI: Oswestry Disability Index
PLIF: Posterior lumbar interbody fusion
Topping-off: Fusion combined with Coflex
VAS: Visual analog scale
